# Supplementary material for: Destructive effects of UVC radiation on Drosophila melanogaster: Mortality, fertility, mutations, and molecular mechanisms
Source: PLoS One. 2024 May 22;19(5):e0303115. doi: 10.1371/journal.pone.0303115 (PMC11111075; doi:10.1371/journal.pone.0303115)
Supplement: S2 File — (DOCX) [file pone.0303115.s005.docx]

**PONE-D-23-25424R3
Destructive Effects of UVC Radiation on Drosophila melanogaster: Mortality, Fertility, Mutations, and Molecular Mechanisms**

**Dear PLOS ONE Editor**
**Thank you for your acceptance requirements e-mail.**

**JOURNAL REQUIREMENTS:**

**1. I included the captions for the Supporting Information files at the end of my manuscript:**

**Files included at the end are the following:**

**Supplementary 1_Mn_SOD qPCR Raw Data_April 11, 2024_Final.xlsx**

**Supplementary 2_Cu_Zn_SOD qPCR Raw Data_April 2024_Final.xlsx**

**Supplementary 3_MTH qPCR Raw Data_April 11, 2024_Final.xlsx**

**2. I included Table 1, in the manuscript file, and it is editable, cell-based objects created using Excel.**

**My regards and best wishes**
