# Supplementary material for: Destructive effects of UVC radiation on Drosophila melanogaster: Mortality, fertility, mutations, and molecular mechanisms
Source: PLoS One. 2024 May 22;19(5):e0303115. doi: 10.1371/journal.pone.0303115 (PMC11111075; doi:10.1371/journal.pone.0303115)
Supplement: Attachment — Submitted filename: PONE-D-23-25424 Review.pdf [file pone.0303115.s006.pdf]

PLOS ONE REVIEW REPORT (PONE-D-23-25424, EMID: 3a5fad0914290368)

Here in this work the authors have seen the effect of UVC rays on *D. melanogaster*. They have done a detailed study on the effect of UV rays on the physiology and morphology of *D. melanogaster*. Further, they have also investigated the underlying molecular mechanism.

Their work is commendable, but similar work has been reported earlier also. This is my suggestion to the authors to discuss briefly, that how this work is different and novel.

The authors argue that the work will help in addressing the potential risks associated with space missions. Though non-ionizing radiations are damaging, it can easily be shielded by space suits. On the other hand, ionizing radiations like X-rays and galactic cosmic radiations are much more difficult to avoid. Effect of ionizing radiation on *D. melanogaster* might be more relevant in such a scenario. May be authors can include such studies in their future work.

The authors assert that reduction in the expression of Mn-SOD gene, Cu-Zn-SOD gene and MTH gene due to UVC radiation in *D. melanogaster* is correlated to the duration of UV exposure. However, in the figure 5, reduction in Mn-SOD expression in the UVC2 group is more than UVC3 group. Similarly, reduction in the expression of Cu-Zn-SOD and MTH in UVC2 group is more than UVC3 group in figure 6 and 7 respectively.

I suggest the authors to explain these phenomena or repeat the experiments to establish their claim that the expression of Mn-SOD, Cu-Zn-SOD and MTH is negatively correlated to UVC exposure in a time dependent manner.
