# Supplementary material for: Destructive effects of UVC radiation on Drosophila melanogaster: Mortality, fertility, mutations, and molecular mechanisms
Source: PLoS One. 2024 May 22;19(5):e0303115. doi: 10.1371/journal.pone.0303115 (PMC11111075; doi:10.1371/journal.pone.0303115)
Supplement: S1 File — (DOCX) [file pone.0303115.s004.docx]

**PONE-D-23-25424R3
Destructive Effects of UVC Radiation on Drosophila melanogaster: Mortality, Fertility, Mutations, and Molecular Mechanisms**

**Dear PLOS ONE Editor**

**Thank you for your acceptance requirements e-mail.**

**JOURNAL REQUIREMENTS:**

1- **I accept the Peer Review History question “opt in”.**
2- **I** **confirm** that the author list and affiliations **are correct on the title page** of my manuscript, and that my author contributions are correct as: **Mohamed Lotfy, Aalaa Khattab, Mohammed Shata, Ahmad Alhasbani, Abdulla Almesmari, Saeed Alsaeedi, Saeed Alyassi, and Biduth Kundu.**

**In details:**

**Mohamed Ibrahim Lotfy:**
Conceptualization
Data curation
Formal analysis
Funding acquisition
Investigation
Methodology
Project administration
Resources
Software
Supervision
Validation
Visualization
Writing – original draft
Writing – review & editing

**Aalaa Khattab:**
Data curation
Formal analysis
Software
Writing – original draft

**Mohammed Shata:**
Data curation
Formal analysis
Investigation
Methodology
Software
Validation
Visualization
Writing – original draft

**Ahmad Alhasbani:**
Data curation
Formal analysis
Investigation
Methodology
Software
Validation
Visualization
Writing – original draft

**Saeed Alsaeedi:**
Data curation
Formal analysis
Investigation
Methodology
Software
Validation
Visualization
Writing – original draft

**Abdulla Almesmari:**
Data curation
Formal analysis
Methodology
Software
Validation
Visualization
Writing – original draft

**Saeed Alyassi:**
Data curation
Formal analysis
Investigation
Methodology
Software
Visualization
Writing – original draft

**Biduth Kundu:**
Data curation
Methodology
Resources
Software

3- **Competing Interests**: **The authors have declared that no competing interests exist.** **This statement is added to the corrected manuscript.**
4- Financial Disclosure: This research received support from an **SDG Grant (2022) awarded by Research Affairs at UAE University, Grant Code: G00004046.** The funders had no role in study design, data collection and analysis, decision to publish, or preparation of the manuscript. **This statement is added to the corrected manuscript.**
5- I checked that the number of Supporting Information captions in my manuscript is matching the number of Supporting Information files uploaded to Editorial Manager before. **Please, in case you still found out mismatching numbers please specify that ones for me.**
6- **I added** “See supplementary qPCR raw data” to the corrected manuscript at the end of figures 5, 6, and 7.
7- I used the Author Formatting Checklist to confirm that your paper meets PLOS ONE's typesetting requirements for References, Tables, and Figures. **Please, inform me if any comments.**

**My regards and best wishes**
