# Supplementary material for: Destructive effects of UVC radiation on Drosophila melanogaster: Mortality, fertility, mutations, and molecular mechanisms
Source: PLoS One. 2024 May 22;19(5):e0303115. doi: 10.1371/journal.pone.0303115 (PMC11111075; doi:10.1371/journal.pone.0303115)
Supplement: Attachment — Submitted filename: 2024 March 3_Response to Reviewers.docx [file pone.0303115.s008.docx]

**'Response to Reviewers'.**

**PLOS ONE REVIEW REPORT (PONE-D-23-25424, EMID: 3a5fad0914290368)**

For the paper entitled “Destructive Effects of UVC Radiation on Drosophila melanogaster: Mortality, Fertility, Mutations, and Molecular Mechanisms.

Dear Editor,

Please, explore the answers the following questions raise by the Editor Reviewers.

Question 1: For Figure number 1,2 & 4 kindly submit proper statistical analysis showing level of significance at 5% and 1% level with software used.

Response:

We modified the paragraph of results in the manuscript “Material and Method Section” as:

The data were expressed as mean ± standard error of the mean. Statistical analysis was conducted using SPSS 15.0 software (IBM Corporation, Armonk, NY, USA), employing one-way ANOVA followed by post-hoc test to determine the significance differences between groups. A significance threshold of P less than 0.05 was set for all analyses. Asterisks (*) indicate significant differences of treated groups compared to the control group.

Moreover, we modified all Figures Legends by addition to all figures the following sentence: Asterisks (*) indicate that the means of the treated groups were significantly different (p<0.05) compared to control group.

Question 2: For morphological assessment, kindly provide one score data against each phenotypic alteration.

Response:

We modified figure 3, by addition of counted histogram as Fig 3 (A) that showed the wings different phenotypic mutations pictures and Fig 3 (B) a quantification showed the percentage of different wings phenotypic mutations in each group. Each experimental group consisted of 40 flies per vial, with an equal distribution of 20 males and 20 females. The histogram showed the percentage of different wings phenotypic mutations. The data is represented as the mean wings mutations percentages ± standard error (SE). Asterisks (*) indicate that the means of the treated groups were significantly different (p<0.05) compared to control group. Each experimental group consisted of 40 flies per vial, with an equal distribution of 20 males and 20 females. As shown in the following:

Question 3: For all the gene expression data kindly provide original gel images of RT PCR as a supplementary file.

Response:

Before we respond to this important question, we are fully confident and totally assure that the reviewers are knowing what we will raise in our coming response.

In our paper, we utilized the Quantitative Real Time PCR (qRT-PCR) Method to assess the relative expression of genes normalized with housekeeping gene. Unlike conventional PCR, qPCR does not yield gel images; instead, it monitors DNA amplification in real-time using fluorescent dyes, with data represented through amplification plots and melting curves. These plots depict fluorescence signal increments over PCR cycles, enabling calculation of cycle threshold (Ct) values that quantify target gene expression. Despite the absence of gel images, qPCR data remain valuable for quantifying gene expression levels and discerning differences between samples, typically presented numerically rather than visually.

Moreover, there are many published papers in PLOS One Journal, presented the Quantitative real-time PCR (qPCR) results as just histograms without gel images such as: Özdaş et al., 2020; Mohan et al., 2016 and Sadi et al., 2015.

1. Özdaş S, Taştekin B, Gürgen SG, Özdaş T, Pelit A, Erkan SO, Tuhanioğlu B, Gülnar B, Görgülü O. Pterostilbene protects cochlea from ototoxicity in streptozotocin-induced diabetic rats by inhibiting apoptosis. Plos one. 2020 Jul 28;15(7):e0228429.
2. Mohan A, Singh RS, Kumari M, Garg D, Upadhyay A, Ecelbarger CM, Tripathy S, Tiwari S. Urinary exosomal microRNA-451-5p is a potential early biomarker of diabetic nephropathy in rats. PloS one. 2016 Apr 21;11(4):e0154055.
3. Sadi G, Baloğlu MC, Pektaş MB. Differential gene expression in liver tissues of streptozotocin-induced diabetic rats in response to resveratrol treatment. PloS one. 2015 Apr 23;10(4):e0124968.
